# CO_2_/N_2_ selectivity with high efficiency using new flexible coordinate organic polymer-based core–shell

**DOI:** 10.1039/d5ra03873a

**Published:** 2025-08-19

**Authors:** Soheila Sharafinia, Nedasadat Saadati Ardestani, Alimorad Rashidi, Fereshteh Abbasy, Pedram Eskandari sabzi

**Affiliations:** a Department of Chemistry, Faculty of Science, Shahid Chamran University of Ahvaz 613578-3151 Ahvaz Iran sharafi.s2014@gmail.com; b Nanotechnology Research Center, Research Institute of Petroleum Industry (RIPI) 14665-1998 Tehran Iran rashidiam@ripi.ir; c School of Energy Engineering and Sustainable Resources, College of Interdisciplinary Science and Technology, University of Tehran 1417935840 Tehran Iran

## Abstract

In this study, organic compounds such as coordinate organic polymers (COPs) and metal–organic frameworks (MOFs) have been investigated as a known technology for CO_2_ and nitrogen (N_2_) adsorption at low and high pressures. The combination of MOFs and COPs is an important innovation in porous materials for gas adsorption and separation. By exploiting the complementary properties of both, this approach improves adsorption performance, stability, and selectivity. This approach reduces the limitations of each material and opens up new opportunities for developing efficient and sustainable adsorbents. These compounds are efficient and cost-effective. First, the COP and COP@ZIF-8 core–shell were synthesized by solvothermal method and then characterized using famous techniques FT-IR, XRD, BET, TEM, HR-TRM, SEM, EDX/Mapping, TGA, and XPS to determine their physicochemical properties. Nanosorbents were tested for adsorption and separation of CO_2_/N_2_. The results showed that the modification of COP with ZIF-8 increased its CO_2_ adsorption capacity from 0.209 to 3.425 mmol g^−1^ at 1 bar and 300.15 K. In addition, the adsorption selectivity of COP@ZIF-8 (20%) and COP@ZIF-8 (30%) core–shells is significantly (207.752 and 200.592 in ambient conditions, respectively) improved compared to pure COP (14.824).

## Abstract 2

In this study, organic compounds such as coordinate organic polymers (COPs) and metal–organic frameworks (MOFs) have been investigated as a known technology for CO_2_ and nitrogen (N_2_) adsorption at pressures of 1–10 bar.

## Graphical abstract

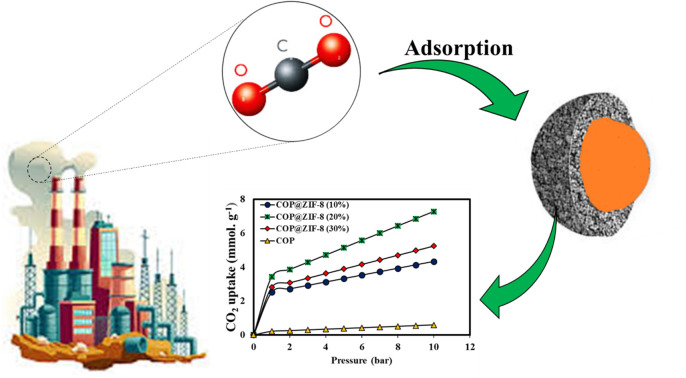

## 1. Introduction

Since the main cause of global warming is carbon dioxide (CO_2_) gas, it is important to absorb/capture, separate and store it sustainably and economically from all its sources and production industries, especially fossil fuel sources.^1–4^ It is important to capture CO_2_ to prevent its excessive emission and to control the overall concentration of greenhouse gases up to the United Nations standard level and finally to reduce the adverse effects on humans and the environment.^5^ Today, various technologies and adsorbent materials, especially the use of solid adsorbents,^6^ such as the utilization of covalent organic framework (COF),^7,8^ MOF adsorbents, mesoporous silica, activated carbon, polybenzimidazole zeolites, mineral materials, and many polymers, have replaced conventional monoethanolamine systems, which were used in most cases.^9–12^ New adsorption technologies with increased adsorption capacity, selectivity, reusability and stability of adsorbent material are of great interest. In the meantime, organic polymers are preferred over other solid adsorbent materials with advantages such as low manufacturing cost and simple manufacturing steps for absorbing various gases in various operational and environmental conditions.^8^ It is noteworthy that the gas adsorption capacity of the solid adsorbent depends on the physical properties of the adsorbents and the structure of their polymer networks, such as the volume of the adsorbent pores, the surface area, and the stiffness and strength of their structure. These physical structural features of the sorbents are guided by the core monomers and their linkers.^13^ As a result, the selection of proper and correct core monomers and linkers is very important. On the other hand, different organic polymer materials that are made from the same building block but are connected by different chain links can have significant differences in physical parameters.^14^ According to the literature results, solid sorbent adsorption systems are based on polymer materials' physical adsorption. The process is acceptable as long as the adsorbent can be reused in the adsorption process. For example, can be referred to the microporous organic polymers (MOPs) with benzene as core monomers and linked by 1,3,5-tris(bromomethyl)-2,4,6-trimethylbenzene agent, that have surface areas (609 m^2^ g^−1^), pore volumes (0.35 cm^3^ g^−1^), and CO_2_ adsorptions of 63.6 mg g^−1^, and for agent of,3,5 tris(bromomethyl)benzene, these values are 688 m^2^ g^−1^, 0.40 cm^3^ g^−1^ and 101 mg g^−1^, respectively.^15^

Recently, a new class of polymer compounds named coordinate organic polymers (COPs) with high capturing capacity of CO_2_ have been presented, which their capturing capacity is related to their physical parameters. These polymers are formed as metal ions in coordination with rigid organic molecules, which are utilized to build multidimensional structures (one-dimensional, two-dimensional, *etc.*)^16^ structures. Liebl *et al.*^17^ have related that the combined functionality of the surfaces and surface area of the network, which may be adjusted and controlled with various linkers, is effective on the capacity of CO_2_ capturing of triazine-based porous polyamide (TPI) substances. Also, in the center of materials such as PTI-1 and PTI-2, the inflexible nature of linkers creates a rigid structure compared to other polymer networks due to the covalent bonds combined with the linkers.^18^ These polymers have a suitable, scalable, capable of bending, and lower-priced synthesis. In the next step, the synthesized COP was used to adsorb and separate CO_2_ and N_2_ gases. Then, to increase the adsorption capacity, ZIF-8 NPs were loaded on COP. It was observed that in the presence of ZIF-8 nanoparticles (NPs), the adsorption and selectivity increased by 12 and 26 times, respectively. The combination of MOFs and COPs is an important innovation in the field of porous materials for gas adsorption and separation. MOFs are known for their very high specific surface area, pore size and function tunability, and ability to host guest molecules. Due to their porous structure and unique properties, these compounds have diverse applications, including adsorption, catalysis, drug delivery, *etc.*^19–24^ At the same time, COPs have significant advantages due to their high chemical stability, structural designability, and surface modification. The combination of these two materials allows for the exploitation of their complementary properties, which leads to improved adsorption performance, increased stability, and higher selectivity in gas separation. This novel approach minimizes the limitations of each material and opens new perspectives in developing efficient and sustainable adsorbents.^25^

## 2. Experimental section

### 2.1. Chemical

All materials used in this work such as poly-vinylpyrrolidone (PVP), hydrofluoric acid (40%, HF), ethanol (99%, EtOH), methanol (99%, MeOH), zinc nitrate hexahydrate (Zn(NO_3_)_3_·6H_2_O), aluminum chloride anhydrous (95%, AlCl_3_), 2-methylimidazole (2-MeIM), benzene (99.5%), 1,2-dichloroethane (99.0%, DCE), dichloromethane (99.5%, DCM), chloroform (99.5%) were purchased from Merck.

### 2.2. Synthesis of COP

**Fig. 1:**
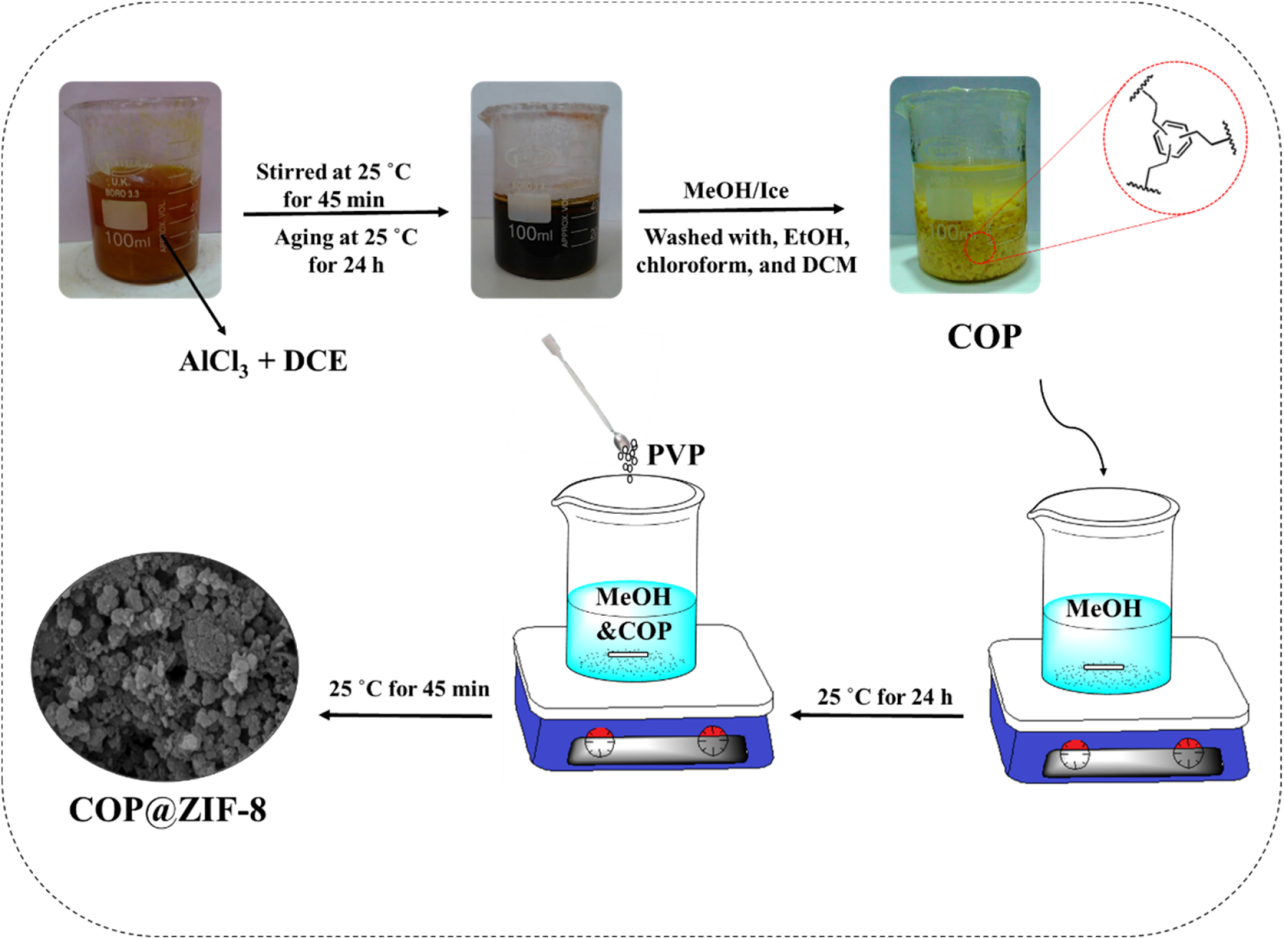
Schematic image of the COP and COP@ZIF-8 synthesis.

For COP synthesis,^16,26^ the solvothermal method was used (Fig. 1). First, 10 g of AlCl_3_ was added to a beaker containing 66.67 mL of DCE and 3.34 mL of benzene and stirred at room temperature for 45 min. Then, the stirring was stopped due to the significant accumulation of particles around the stirring bar. The mixture was aged for 24 h. The reaction mixture was quenched by mechanically breaking the aggregate pieces and slowly adding the MeOH/ice mixture (66.67 mL). The resulting mixture was filtered, and the precipitate obtained was stirred twice with distilled water (85 °C, 4 h), four times with EtOH (60 °C, 6 h), four times with chloroform (60 °C, 6 h), and two times with DCM (25 °C, 6 h). The yellow powder was transferred to a vacuum oven and dried at 100 °C.

### 2.3. Synthesis of the COP@ZIF-8

In a beaker containing 19 mL pure MeOH, 100 mg COP was added and sonicated at room temperature for 1 h (Fig. 1). Then, 20 mg of PVP was dispersed in the resulting mixture and stirred for 12 h under ambient temperature. Next, mg of Zn(NO_3_)_3_·6H_2_O (200 mg) was added to the above. Thereafter, 8 mL of MeOH solution, including 250 mg of 2-MeIM, was added to the combination and agitated for 45 min. The resulting mixture was centrifuged for 5 min at a rate of 9000 rpm. Eventually, the solid samples were washed with MeOH (30 mL, 3×) and 12 h dried in an oven at 90 °C.^26^ This sample is 20 wt%. The synthesis method of other composite samples with different amounts of ZIF-8 (10 and 30 wt%) on COP was similar to the preparation method of (20 wt%), and only the ratio of raw materials (amount of Zn(NO_3_)_2_·6H_2_O and 2-methylimidazole) was adjusted according to the desired weight percentage. Other steps were performed similarly, including sonication, stirring, centrifugation, washing, and drying.

### 2.4. Experimental setup

**Fig. 2:**
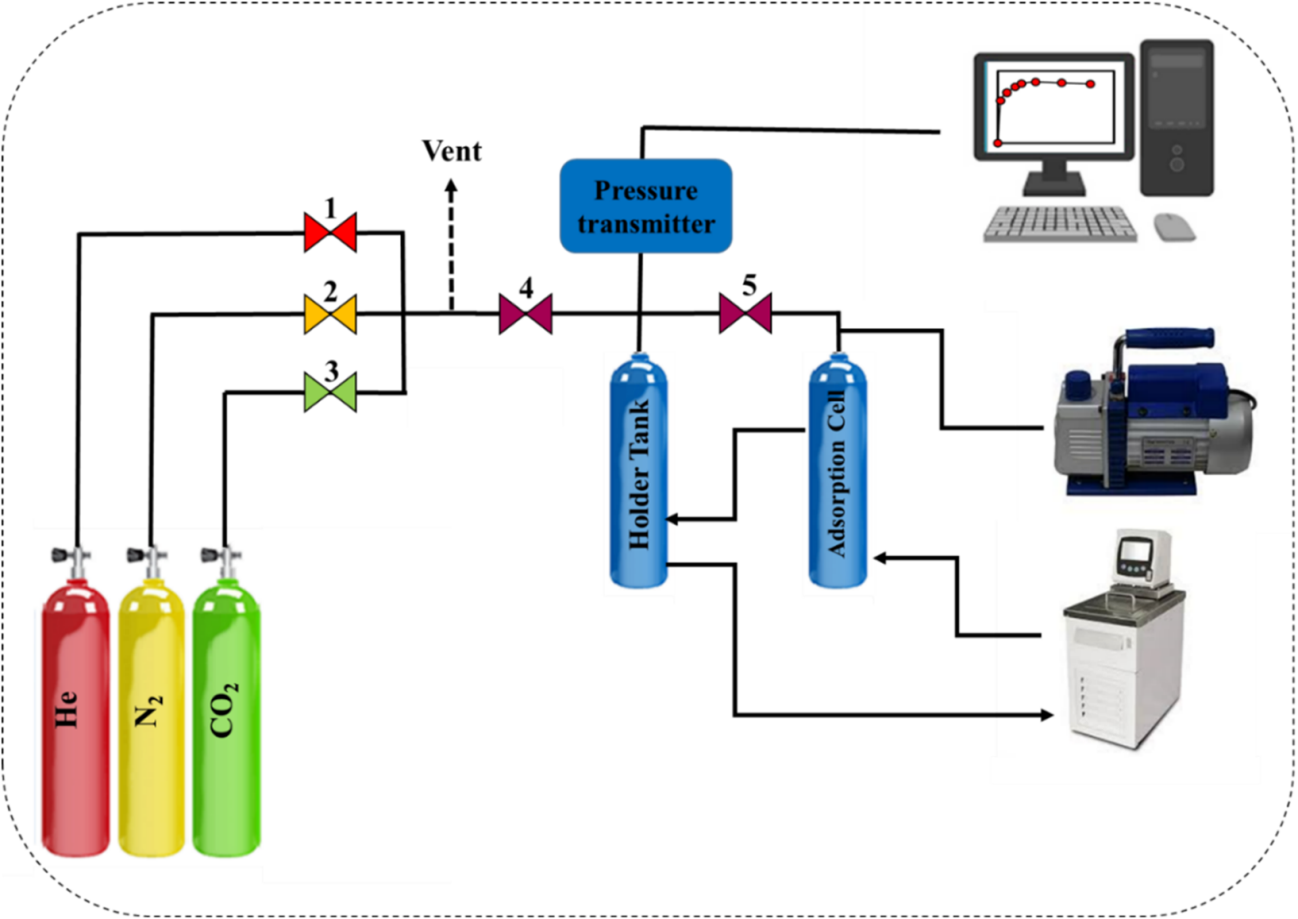
Schematic image of the in-house build gas adsorption set-up.

Fig. 2 represents the schematic of the in-house volumetric setup for gas adsorption. This unique setup became capable of measuring the equilibrium adsorption potential of gases such as CO_2_ and N_2_ at 1–10 bar and different temperatures. First, the weighed adsorbent was transferred to the adsorption cell and completely sealed. The adsorption cell was tested with high-pressure (≥50 bar) helium gas. Then, the adsorbent was degassed using the pressure–temperature swing method (200 degrees Celsius and vacuum pressure) to remove the undesirable molecules adsorbed from the atmosphere. A vacuum pump, a heater, and a temperature controller provided the vacuum pressure and temperature of the adsorption cell, respectively. After 4 h, the heater was turned off, the pump was vacuumed, and the cell was cooled down to the adsorption temperature with a smooth ramp. A circulator kept the temperature of the gas and the adsorption cell constant during the adsorption process. To measure the equilibrium adsorption capacity of the gas on the weighted adsorbent, the pressure of the gas cell was set to the desired pressure by opening V-3 and V-4, but V-5 was closed. Then, by opening V-5, the desired gas enters the absorbed cell, and the pressure of the gas cell decreases. The constant pressure of the adsorption cell represents the state of equilibrium, and the difference between the initial and equilibrium pressures observed is the basis for volumetric adsorption calculations. The pressure drop is due to the gas adsorption on the surface and the dead volume of the setup. Since helium molecules are not adsorbed on the adsorbent, helium adsorption was performed to reduce the dead volume from the calculations. The total CO_2_ or N_2_ adsorption amount was calculated using the eqn (S1)–(S5).

## 3. Results and discussion

In this study, composite materials with different weight ratios of ZIF-8 (10, 20, and 30 wt%) were synthesized on COP to investigate the effect of ZIF-8 content on carbon dioxide absorption. After the adsorption experiments, the sample containing 20 wt% ZIF-8 showed the best performance in terms of CO_2_ absorption and was selected as the optimal sample. Therefore, all characterization analyses (including FT-IR, XRD, BET, TEM, HR-TRM, SEM, EDX/Mapping, TGA, and XPS) were performed on this optimal sample to investigate its structural and chemical properties accurately.

### 3.1. Characterization

**Fig. 3:**
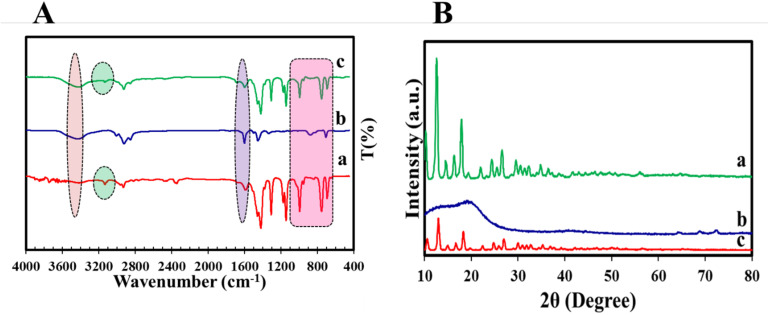
(A) FT-IR results of (a) ZIF-8 NPs, (b) COP, (c) COP@ZIF-8; (B) XRD of (a) ZIF-8 NPs, (b) COP, (c) COP@ZIF-8.

The FT-IR spectra of COP, ZIF-8 NPs, and COP@ZIF-8 are depicted in Fig. 3A. All the peaks observed in these samples are detailed in Table 1. Upon comparing the FT-IR spectra of the COP@ZIF-8 core–shell with those of pure ZIF-8 NPs, distinct peaks are observed that correspond to aromatic C–H stretching at 385 cm^−1^, aromatic C–C stretching at 1600 cm^−1^, and aromatic C–H bending characteristic of COP within the range of 600–900 cm^−1^.

**Table 1:** Results of surface chemistry analysis using FT-IR

Sample	Function groups	This work	Ref.
COP	Aromatic C–H bending	600–900	28
	Aliphatic C–H bending	1000–1200	28
	Aromatic C–C stretching	1400–1600	28
	C–H stretching	<3000	28
	Aromatic C–H stretching	>3000	28
ZIF-8 NPs	C–N in the imidazole ring	993	29
	C=N in the imidazole ring	1587	29
	C–H stretching of –CH_3_	2963	30
	C–N (stretching)	3134	30
COP@ZIF-8	C–N in the imidazole ring and aromatic C–H bending	993	29
	C=N in the imidazole ring and aromatic C–C stretching	1457	29
	Aromatic C–C stretching	1599	28
	C–H stretching of –CH_3_	2927	30
	C–N (stretching)	3133	30
	Aromatic C–H stretching	3433	28

The XRD patterns for COP, ZIF-8 NPs, and COP@ZIF-8 are shown in Fig. 3B. The XRD analysis of ZIF-8 NPs (Fig. 3B-a) reveals high crystallization. The appearance of planes 002, 112, 022, 013, and 222 in this sample corresponds to peaks observed at 2*θ* values of 10.35°, 12.70°, 14.80°, 16.40°, and 18°, respectively.^27^ These values are consistent with those reported in the literature. The XRD pattern in Fig. 3B-b shows a broad peak at 2*θ* values of 22° (002),^28^ indicating the presence of graphitic carbon and confirming the amorphous structure of COP. Also, the appearance of several small peaks in this pattern indicates that alumina is present in the COP structure. Upon comparing the XRD patterns of COP@ZIF-8 with those of pure ZIF-8, it is clear that the peak intensities are lower, which can be attributed to the reduced crystallinity caused by the presence of amorphous COP within the composite.

**Fig. 4:**
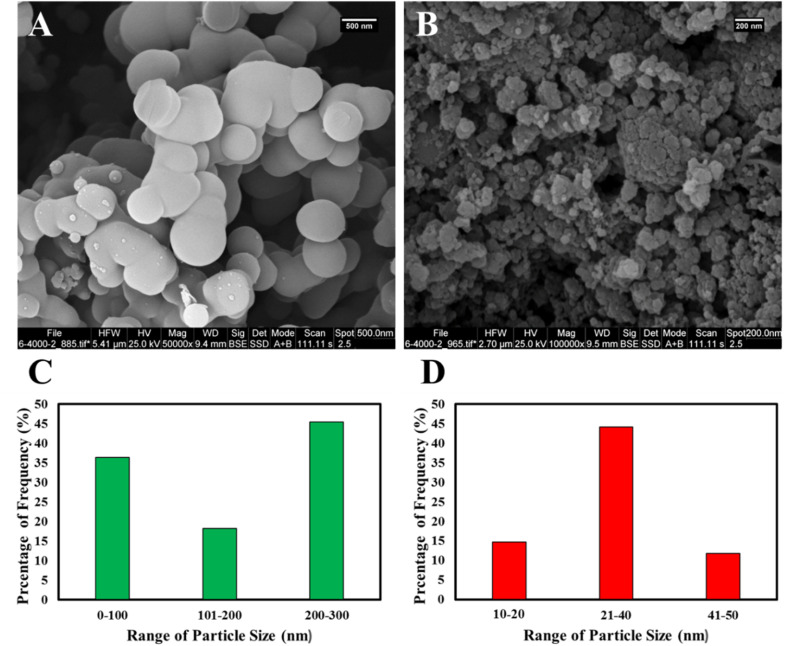
SEM images of (A) COP and (B) COP@ZIF-8; PSD results of (C) COP and (D) COP@ZIF-8.

**Fig. 5:**
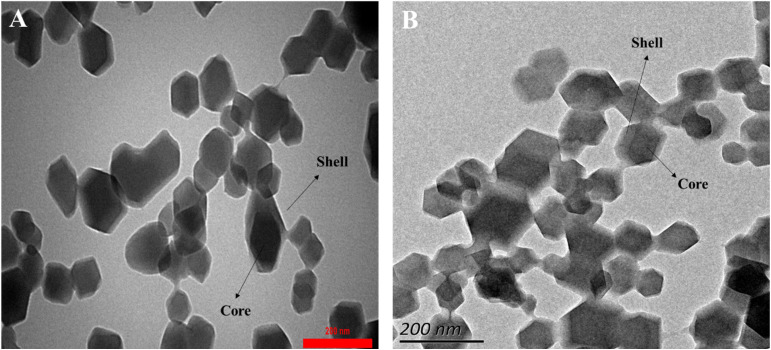
(A) TEM and (B) HR-TEM images COP@ZIF-8.

One of the crucial aspects in determining the quality of materials is their surface morphology. Surface morphology, which includes features such as texture, roughness, and microscopic structure of the material surface, plays a decisive role in key properties such as adsorption capacity, catalytic activity, and mechanical stability; these properties are recognized as key indicators of material quality.^31^ Therefore, understanding and controlling surface morphology is a fundamental step in optimizing the performance and improving the quality of materials. The COP@ZIF-8 core–shell structure is confirmed by the SEM, TEM, and HR-TEM images shown in Fig. 4 and 5, respectively. Fig. 4A illustrates the spherical structure of COP. In the SEM image of the COP@ZIF-8 sample (Fig. 4B), the rough surface and uniform distribution of ZIF-8 NPs on the COP surface are observed, indicating the successful loading of NPs on the polymer matrix. This porous and rough surface structure can help increase the active surface area and improve the adsorption properties. According to the particle size distribution (PSD) plots, the maximum size of COP is between 200–300 nm, and by loading ZIF-8 NPs, the particle size reaches 21–40 nm due to the presence of these NPs (Fig. 4C and D). Loading of ZIF-8 NPs onto the COP matrix leads to structural and morphological changes on the composite surface, which explains the observed particle size reduction. This size reduction is due to increased strong interfacial interactions between ZIF-8 NPs and COP polymer chains, which prevent particle aggregation and provide a more homogeneous dispersion. In addition, the porous structure and very high specific surface area of ZIF-8 act as a dispersing agent, causing the breakup of larger COP particle aggregations. In other words, the reduction in particle size after ZIF-8 loading is due to the effect of modulators on the crystal growth rates, which leads to the formation of smaller particles and better dispersion. This phenomenon has been reported in several studies, which show that the loading of MOFs, especially ZIF-8, can improve the particle size distribution and increase the active surface area of the composites, which in turn leads to improved functional properties such as adsorption and catalysis.^32^

Also, TEM (Fig. 5A) and HR-TEM (Fig. 5B) images corroborate the core–shell formation of the COP@ZIF-8 structure. The central part of the particles, which is seen as denser and with higher contrast, constitutes the core, which is made of the COP material. Around this core, a uniform layer with a different contrast, known as the shell, is observed, which is made of ZIF-8 NPs. This layered structure and the clear separation between the core and the shell indicate the successful loading of ZIF-8 NPs onto the COP. These results confirm that the ZIF-8 NPs are uniformly coated on the COP surface, and this core–shell structure can help improve the physical and chemical properties of the composite.

**Fig. 6:**
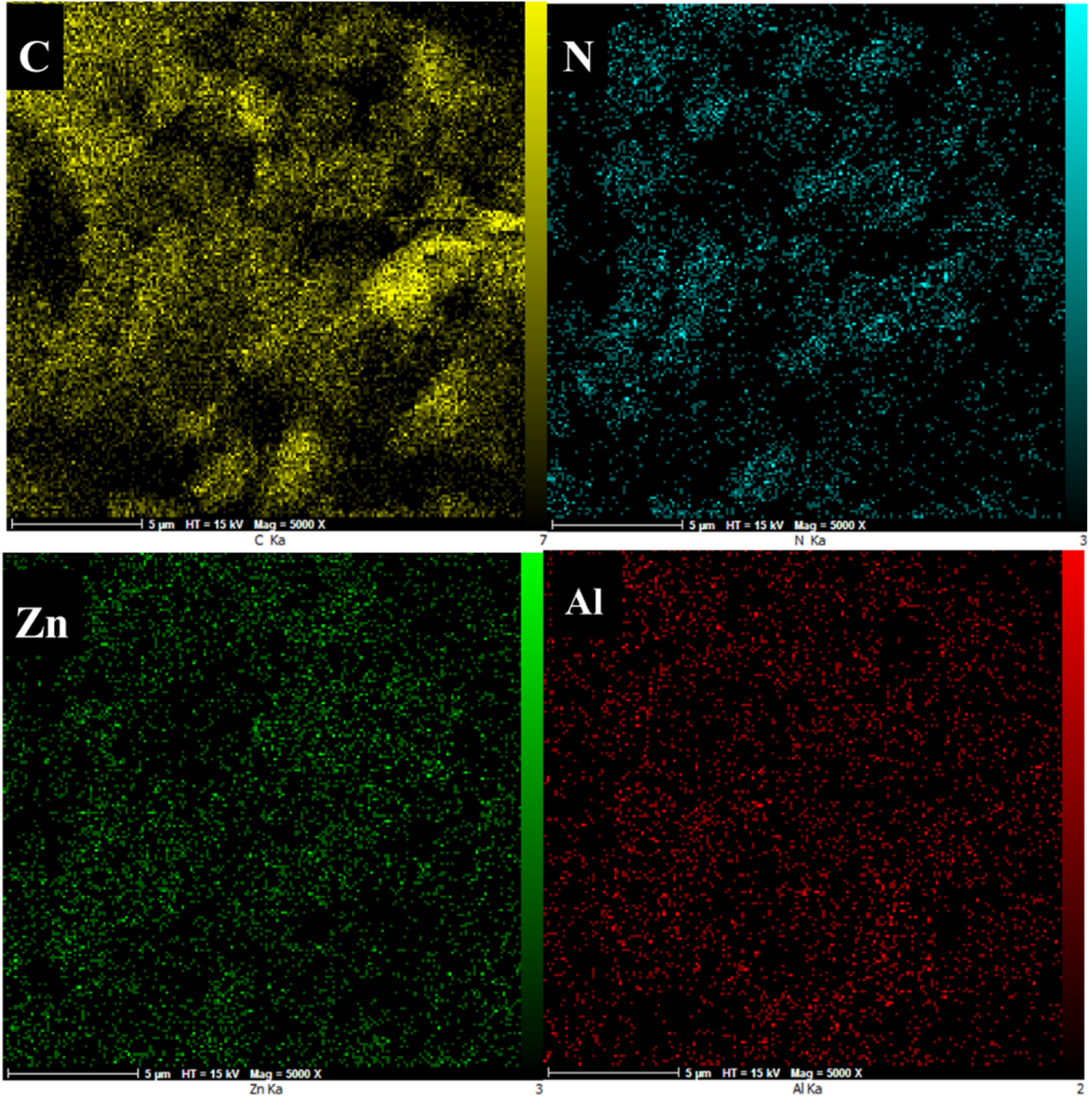
Elemental mapping of COP@ZIF-8.

Based on the elemental mapping presented in Fig. 6, the distribution of Al and Zn elements in the COP@ZIF-8 structure is uniform and homogeneous. The aluminum element is related to the COP matrix, while the Zn element indicates the successful loading of ZIF-8 NPs into the composite structure. This uniform distribution of both elements confirms the proper dispersion of NPs and the structural integrity of the sample, which can affect the improvement of the material's physical and chemical properties.

**Fig. 7:**
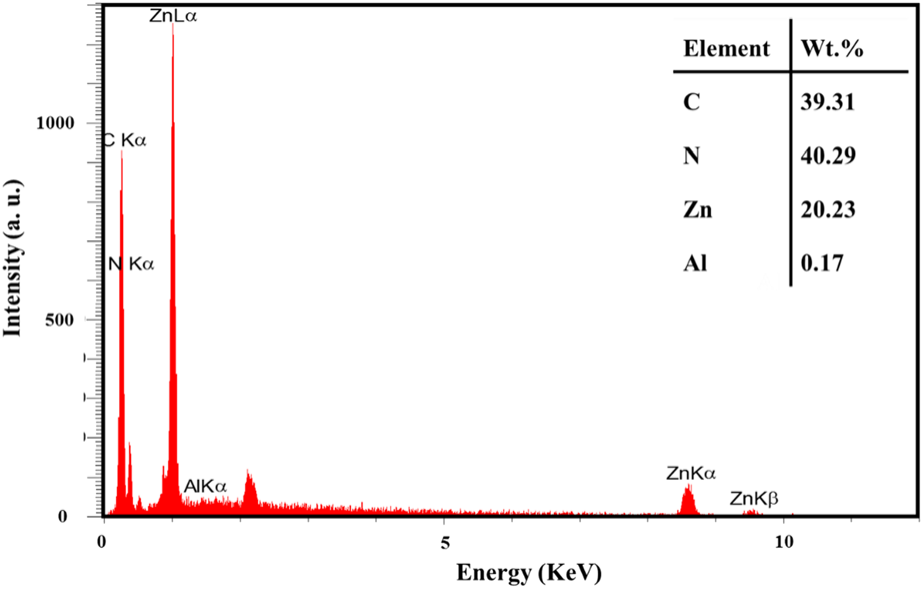
EDX results of COP@ZIF-8.

EDX analysis is a powerful method for identifying and determining the distribution of chemical elements in samples. This study used EDX to demonstrate the presence and uniform distribution of Al and Zn elements in the COP@ZIF-8 structure (Fig. 7). This analysis shows that ZIF-8 NPs were successfully loaded onto the COP matrix, and the distribution of elements was homogeneous throughout the sample. Therefore, the EDX results confirm the structural integrity and proper dispersion of NPs in the composite, which can positively affect the material's functional properties.

**Fig. 8:**
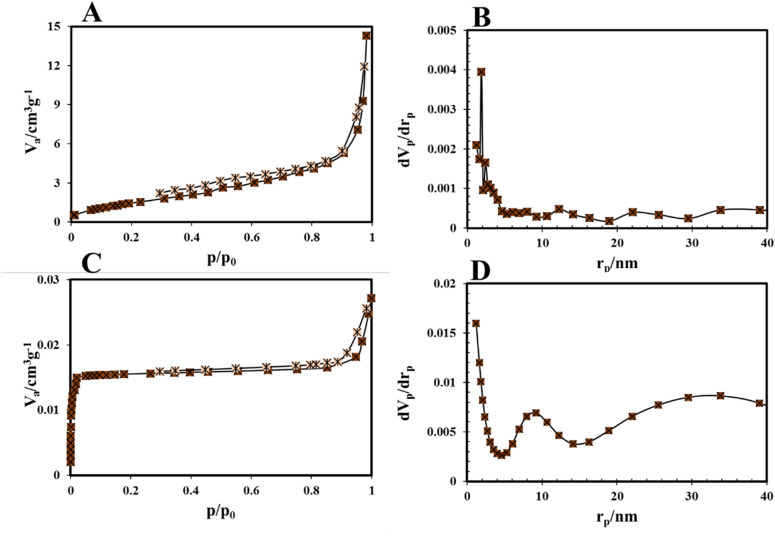
(A) Nitrogen adsorption/desorption isotherm, and (B) BJH plots of COP; (C) nitrogen adsorption/desorption isotherm, and (D) BJH plots of COP@ZIF-8.

BET analysis was performed accurately and in accordance with the methods presented in the SI to determine the specific surface area and adsorption properties of the sample. The surface area values for COP and the COP@ZIF-8 core–shell were determined to be 7.845 m^2^ g^−1^ and 1356.942 m^2^ g^−1^, respectively. As observed, the pore diameter decreases with the formation of core–shell. According to the standard IUPAC classification, pore sizes are divided into three categories, including micropores (<2 nm), mesopores (2 to 50 nm), and macropores >50 nm.^16,33–35^ COP with 2–50 nm pore sizes, are classified as mesoporous. According to the IUPAC classification, the adsorption isotherm of COP@ZIF-8 (Fig. 8C) is of type I. It has a hysteresis loop of type H4, indicating the presence of a combined micro- and mesoporous structure in this adsorbent.^36–38^ The presence of micropores could be demonstrated from the rapid increase of gas adsorption at relative low pressure. Meanwhile, the rapid upturn and hysteresis loop at relative high pressure suggests the presence of mesopores.^39^ A more detailed analysis of the adsorption–desorption isotherms shows that the studied COP@ZIF-8, besides having a mesoporous structure, also contains a significant percentage of microporosity; this is entirely consistent with the high values of the reported specific surface area. Therefore, the classification of these materials is not limited to being mesoporous only, and their structure is a combination of microporous and mesoporous networks, which can provide more optimal adsorption and transport properties. The shape and type of the adsorption–desorption isotherm usually indicate the type and size of the pores. Parts of the isotherm corresponding to adsorption at low relative pressures indicate the presence of micropores, while adsorption at medium to high relative pressures indicates mesopores. Therefore, a detailed analysis of the isotherm curve confirms the composition of micro- and mesopores.

The adsorption isotherm of COP-150 (Fig. 8A) is of type III according to the IUPAC classification, indicating weak interactions between the adsorbate and the adsorbent surface, as well as monolayer to multilayer adsorption on non-porous or coarsely porous surfaces. This type of isotherm is usually observed when the adsorption is mainly due to interactions between adsorbate molecules and the adsorbent surface lacking strong adsorption sites.^26^ The observed hysteresis loop is of type H3, indicating the presence of slit-shaped pores or aggregation of plate-shaped particles that do not limit adsorption at high partial pressures. According to the IUPAC standard, type H3 hysteresis is usually observed in materials with mesoporous porous structures and specific pore geometries, which are consistent with the structure of zeolites and similar materials such as COP-150.^40,41^ Based on the BJH (Barrett–Joyner–Halenda) plots shown in Fig. 8B and D, the particle distribution falls within the range of 2–50 nm, confirming that the particles are mesoporous. Table 2 summarizes the essential parameters associated with the porosity of the COP and COP@ZIF-8 samples, including total pore volume, surface area, pore diameter, and BJH values.

**Table 2:** Porous structure parameters of COP, and COP@ZIF-8

Adsorbent	*S*_BET_ (m^2^ g^−1^)	Mean pore diameter (nm)	Pore volume^BJH^	*V*_*t*_ (cm^3^ g^−1^)
COP	7.84	15.11	0.05	0.02
COP@ZIF-8	1356.94	2.13	0.43	0.73

**Fig. 9:**
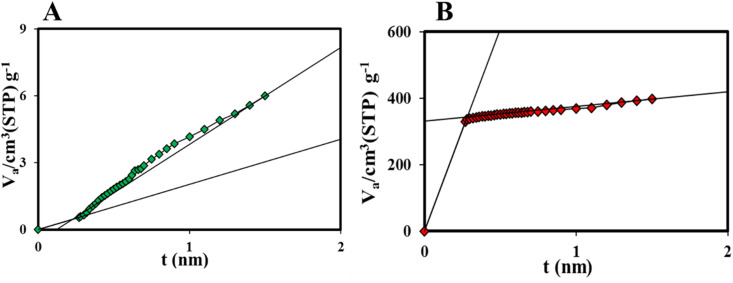
*t*-plot curves of (A) COP, (B) COP@ZIF-8 core–shell.

Fig. 9A shows *t*-plot of COP. The external surface area (*S*_ext_) of the COP sample was 6.70 m^2^ g^−1^. A good correlation was observed between the total surface area determined by the BET method (7.84 m^2^ g^−1^) and the sum of the external and mesosphere surface areas obtained through the *t*-plot (6.70 m^2^ g^−1^). The mesosphere surface area (*S*_mes_) is calculated by subtracting the external surface area from the total surface area (*S*_BET_). In other words, the *S*_mes_ equals the difference between the *S*_BET_ and the *S*_ext_ determined by the *t*-plot. For the COP sample, the *S*_mes_ was obtained to be 1.14 m^2^ g^−1^.^42^ The external surface area was first determined from the *t*-plot to calculate the micropore surface area of COP@ZIF-8. The use of the *t*-plot method for calculating the surface area of micropore (*S*_mic_) materials is discussed, and only the calculation of the external surface area using the high-pressure (or high layer thickness) fitting is recommended. The *t*-plot of COP@ZIF-8 was determined (Fig. 9B), and *S*_ext_ was calculated for COP@ZIF-8 (67.22 m^2^ g^−1^). The total surface area from the BET method is 1356.94 m^2^ g^−1^. The *S*_mic_ of COP@ZIF-8 was then obtained by the difference between *S*_BET_ and *S*_ext_,^42^ which gave a value of *S*_mic_ = 1289.72 m^2^ g^−1^.

According to the results of TGA analysis, which show extraordinary thermal stability for adsorbent materials, including COP and COP@ZIF-8 core–shell at temperatures above 500 °C, these nanosorbents can be classified as the most stable porous materials (Fig. S1-A-b). The significant peaks of TGA analysis were related to the decomposition of ZIF-8, which occurred because of the burning of organic linker molecules in the framework.^43^ This phenomenon has led to the formation of zinc oxide, or the final product of ZIF-8 NPs.^43^ As shown in Fig. S1-A-a, the decomposition of Al^3+^ has occurred with a decrease in the weight of COP at a temperature of 445 °C. The first weight loss occurred at a temperature lower than 100 °C, related to the loss of water or residual/remaining evaporation,^44^ evident in Fig. S1-A-b. Also, the second decomposition at a temperature of 230 °C is related to removing oxygenated functional groups from the surface and destroying volatile substances in the sample.

**Fig. 10:**
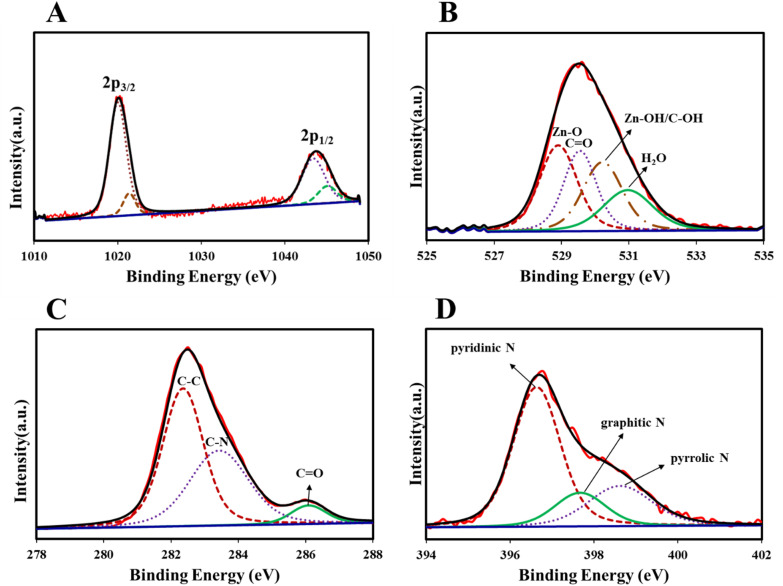
XPS spectra of COP@ZIF-8 core–shell: (A) Zn 2p, (B) O 1s, (C) C 1s, (D) N 1s.

The XPS survey spectrum shows four prominent peaks of C 1s, N 1s, O 1s, and Zn 2p (Fig. S1-B). Also, Table 3 reports the C, N, Zn, and O content of COP@IF-8 core–shell. The sharp Zn 2p spectrum confirms the presence of two different chemical states of Zn, including 61.1% of Zn 2p_3/2_ and 38.9% of Zn 2p_1/2_ (Fig. 10A), which indicate interactions of Zn with carbon atoms in different chemical environments or with different oxidation states. The high-resolution O 1s spectrum confirmed the presence of four different carbon groups, including 32.4% of Zn–O, 24.5% of C=O, 28.3% of Zn–OH/C–OH, and 14.8% of H_2_O (Fig. 10B). The high-resolution C 1s spectrum was fitted by four peaks, suggesting the existence of three carbon species on the surface of the sample: 51.95% of C–C, 41.98% of C–N (28.25%), 6.06 of C=O (Fig. 10C). The high-resolution N 1s spectrum was fitted by three peaks suggesting the existence of nitrogen species on the surface of the sample: 54.6% of pyridinic N, 20.62% of graphitic N, and 24.78% of pyrrolic N (Fig. 10D).

**Table 3:** The elemental concentration of surface functional groups derived from XPS data of NPC@ZIF-8 core–shells

	Compound	Peak position (eV)	Concentration (%)
Zn 2p	Zn 2p_3/2_	1020.2–1021.5	61.1
	Zn 2p_1/2_	1043.3–1045.2	38.9
O 1s	Zn–O	528.9	32.4
	C=O	529.3	24.5
	Zn–OH/C–OH	529.7	28.3
	H_2_O	531.2	14.8
C 1s	C–C	282.3	51.95
	C–N	283.4	41.98
	C=O	286.04	6.06
N 1s	Pyridinic N	396.6	54.6
	Graphitic N	397.5	20.62
	Pyrrolic N	398.56	24.78

### 3.2. CO_2_ and N_2_ adsorption performance

**Fig. 11:**
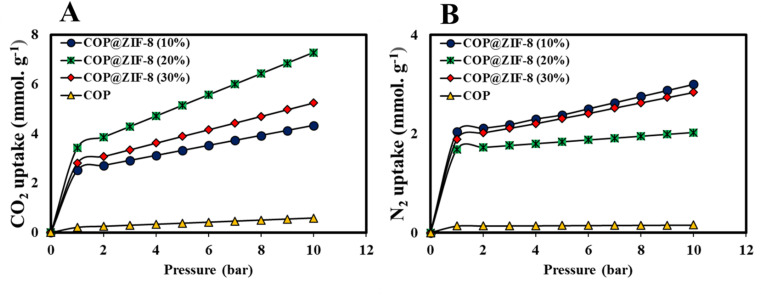
(A) CO_2_ adsorption, and (B) N_2_ adsorption of COP and COP@ZIF-8 (10–30%) at 300.15 K and up to 1–10 bar.

A volumetric in-house setup (Fig. 2) was used to measure the equilibrium uptake capability of CO_2_ and N_2_ on COP, COP@ZIF-8 (10–30%) samples at 0–10 bar and 300.15 K, and the results are indicated in Fig. 11. As may be observed from Fig. 11A, COP has the bottom CO_2_ adsorption capability inside the variety of 0–10 bar because its porosity and surface area are lower than the ones of other nanosorbents. At low pressures, due to the integration of ZIF-8 NPs with COP, an increase in CO_2_ adsorption capacity is observed. It can be said that the presence of amine functional groups in the pores of COP@ZIF-8 samples strengthens the interaction between the nanosorbent surface and the CO_2_ molecule. Since the polarizability of CO_2_ is high (26.3 × 10^−25^ cm^3^),^45–50^ it increases the electrostatic attraction towards the amine-containing surface groups of COP@ZIF-8 nanosorbents and thus improves CO_2_ adsorption. In addition, by increasing the ZIF-8 NPs mass ratio from 10–30%, the CO_2_ adsorption capacity increased significantly (2.509–3.425 mmol g^−1^ at 1 bar and 300.15 K). Fig. 11B shows the N_2_ adsorption isotherms of the nanosorbents. COP@ZIF-8 (10%) has the highest N_2_ adsorption capacity, followed by COP@ZIF-8 (30%), COP@ZIF-8 (20%), and COP, which is because the uptake loading of N_2_, as an inert gas, is mainly affected by the textural properties of the adsorbent (*i.e.*, the higher the porosity, the higher the N_2_ adsorption capacity). The low adsorption value of N_2_ by the surface of COP and synthesized nanocomposites is due to its inert nature (*i.e.*, the polarizability of 17.6 × 10^−25^ cm^3^),^45,51^ which prevents its interaction with the surface of the absorbers.

On the other hand, the surface area of the COP sample before ZIF-8 loading was about 7.84 m^2^ g^−1^, and its pore volume was 0.02 cm^3^ g^−1^ with an average pore diameter of 15.11 nm, indicating a mesoporous structure with relatively large pores. After ZIF-8 loading, the surface area decreased to 1356.94 m^2^ g^−1^, the pore volume to 0.73 cm^3^ g^−1^, and the pore diameter to 2.13 nm. These changes indicate a modification of the porous structure towards finer and more active micro-mesopores, which significantly increases the CO_2_ adsorption capacity. Also, in terms of molecular geometry, both N_2_ and CO_2_ are linear molecules, but CO_2_ has a greater ability to penetrate and adsorb in finer pores due to its smaller effective diameter (∼0.33 nm) and higher polarity. In comparison, N_2_, with an effective diameter of about 0.364 nm, tends to adsorb in larger pores. Therefore, reducing pore diameter to about 2.13 nm after ZIF-8 loading provides optimal conditions for selective adsorption of CO_2_ over N_2_. Also, the significant increase in specific surface area and pore volume increases the active surface area for the adsorption of gas molecules, and the optimal distribution of pore sizes (micro and mesoporous) facilitates the diffusion and storage of gases. Therefore, in addition to the polarization parameters, other parameters such as pore volume, pore size and shape, and specific surface area play a key role in explaining and improving the adsorption performance of N_2_ and CO_2_, and this relationship is visible in the BET data and corresponding analyses.^52,53^

As can be seen from the figure, for CO_2_ adsorption, the sample with 20% ZIF-8 loading showed the best performance with adsorption of about 8 mmol g^−1^ due to the optimal balance between increasing the specific surface area and maintaining good pore accessibility. Lower loading (10%) may not provide sufficient specific surface area, and higher loading (30%) may cause pore blockage and reduced permeability, which limits adsorption.^53^ In contrast, for N_2_ adsorption, the sample with 10% loading was optimal because the N_2_ molecule requires more open pores and easier access due to its different size and geometry and weaker interactions with the adsorbent surface. Higher loading may narrow the pores and limit N_2_ penetration, so optimal adsorption is achieved at lower loading percentages.^40^

The significant change in the slope of the CO_2_ and N_2_ adsorption isotherms on COP@ZIF-8 can be explained by two main factors: the microporous structure of the material and the heterogeneity of the adsorption sites on its surface. Initially, the adsorption of gases occurs mainly at sites with high adsorption energy or in more accessible micropores. This part of the adsorption occurs in the low-pressure range, at a relatively gentle rate and continuously, because gas molecules initially prefer to settle at these high-energy sites. As the pressure increases and these primary sites become saturated with molecules, the adsorption shifts to secondary sites or pores that have lower adsorption energy or are more restricted in accessibility. This change in the adsorption mechanism causes a noticeable and sudden increase in the slope of the isotherm, which appears as a sharp change in the diagram. This behavior is widespread and well-known in microporous materials such as ZIF-8 and similar materials from the MOF family, which have a multi-level porous structure and heterogeneous adsorption energy distribution. Moreover, in the case of CO_2_, this effect is generally more pronounced because this molecule has a higher affinity and energy of interaction with the adsorbent surface than N_2_. Therefore, the difference in the rate and mechanism of adsorption at different sites is more obvious. Numerous studies in the scientific literature have confirmed such phenomena and have shown that this type of isotherm behavior is due to the coincidence of the microporous structure of the material and the energy heterogeneity of the adsorption sites. Therefore, this sudden change in the slope of the isotherm is not just a hypothesis but a reliable phenomenon well supported by experimental data and reliable scientific reports.^54–56^

To better investigate the adsorption behavior of N_2_ and CO_2_ and the adsorbents used, the experimental data were evaluated using Langmuir (eqn (1)), Langmuir–Freundlich (eqn (2)), Dual-Site Langmuir (DSL) (eqn (3)), and Dual-Site Langmuir–Freundlich (DS-LF) (eqn (4)) equations. The regression coefficient (*R*^2^) and parameters of these isotherms are presented in Tables 4 and 5. The DSL model had excellent accuracy for all samples (Fig. S2 and S3).1
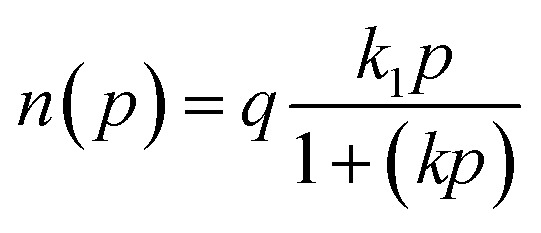
2
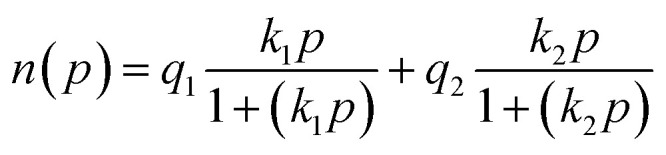
3
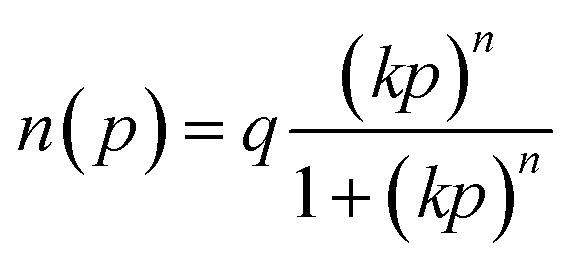
4
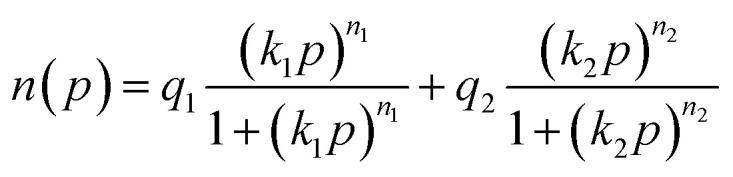
in these equations, *P* is the total gas pressure in the bar, *q* is maximum adsorption capacity or single layer capacity of the adsorbent in mmol g^−1^, and *n*(*p*) is the adsorption capacity in mmol g^−1^. *q*_1_, and *q*_2_ are the maximum adsorption capacities (mmol g^−1^) corresponding to two different types of adsorption sites and are not necessarily equal and should not be considered the same. Also, *k*_1_ and *k*_2_ are the adsorption coefficients or affinity constants (1 bar^−1^), and *n*_1_ and *n*_2_ are parameters indicating the deviation from the ideal homogeneous surface.

**Table 4:** The extended isotherm parameters for N_2_ at 300.15 K and 1–10 bar pressure

Isotherms	Parameters	Adsorbent			
		COP	COP@ZIF-8 (10%)	COP@ZIF-8 (20%)	COP@ZIF-8 (30%)
Langmuir	*q* (mmol g^−1^)	0.152	2.816	1.963	2.272
	*k* (1 bar^−1^)	6.105	1.825	4.652	1.692
	*R* ^2^	0.997	0.994	0.998	0.995

DSL	*q*_1_ (mmol g^−1^)	0.132	804.573	12.094	325.642
	*k*_1_ (1 bar^−1^)	10 000	0.0001	0.003	0.0003
	*q*_2_ (mmol g^−1^)	1.149	1.884	1.644	1.802
	*k*_2_ (1 bar^−1^)	0.002	10 000	10 000	163.862
	*R* ^2^	1	0.999	1	0.999

LF	*q* (mmol g^−1^)	0.720	123.383	14.838	113.425
	*k* (1 bar^−1^)	27.142 × 10^−9^	8.533 × 10^−11^	1.868 × 10^−10^	1.952 × 10^−10^
	*n*	0.079	0.179	0.093	0.185
	*R* ^2^	0.998	0.998	0.999	0.998

DS-LF	*q*_1_ (mmol g^−1^)	0.708	7.356	2.509	2.852 × 10^−24^
	*k*_1_ (1 bar^−1^)	0.003	3.989 × 10^−11^	0.021	1.394 × 10^−13^
	*n*_1_	1.030	0.014	1.147	3.531 × 10^−12^
	*q*_2_ (mmol g^−1^)	0.133	3.290 × 10^−28^	3.310	10.441
	*k*_2_ (1 bar^−1^)	7647.160	1.366 × 10^−20^	1.987 × 10^−30^	0.001
	*n*_2_	1.585	3.989 × 10^−11^	4.476 × 10^−7^	0.230
	*R* ^2^	0.999	0.997	0.998	0.998

**Table 5:** The extended isotherm parameters for CO_2_ at 300.15 K and 1–10 bar pressure

Isotherms	Parameters	Adsorbent			
		COP	COP@ZIF-8 (10%)	COP@ZIF-8 (20%)	COP@ZIF-8 (30%)
Langmuir	*q* (mmol g^−1^)	0.766	4.195	7.394	5.151
	*k* (1 bar^−1^)	0.224	1.060	0.597	0.839
	*R* ^2^	0.991	0.993	0.991	0.992

DSL	*q*_1_ (mmol g^−1^)	0.167	804.573	518.031	121.884
	*k*_1_ (1 bar^−1^)	10 000	0.0001	0.0008	0.002
	*q*_2_ (mmol g^−1^)	24.368	1.884	2.992	2.522
	*k*_2_ (1 bar^−1^)	0.001	10 000	10 000	10 000
	*R* ^2^	0.999	0.999	1	0.999

LF	*q* (mmol g^−1^)	352.850	456.456	1862.090	832.554
	*k* (1 bar^−1^)	2.240 × 10^−7^	6.430 × 10^−10^	8.713 × 10^−9^	1.706 × 10^−9^
	*n*	0.496	0.249	0.344	0.286
	*R* ^2^	0.997	0.998	0.997	0.998

DS-LF	*q*_1_ (mmol g^−1^)	0.300	7.356	22.779	22.075
	*k*_1_ (1 bar^−1^)	1.339	0.014	0.027	0.016
	*n*_1_	6.984	0.254	1.304	1.115
	*q*_2_ (mmol g^−1^)	0.031	3.290 × 10^−28^	10.301	5.162
	*k*_2_ (1 bar^−1^)	0.280	1.366 × 10^−20^	7.471 × 10^−5^	1.589 × 10^−16^
	*n*_2_	1.962	3.989 × 10^−11^	0.083	4.121 × 10^−16^
	*R* ^2^	0.998	0.997	0.999	0.998

Fitting N_2_ adsorption data with different isotherm models (Table 4) shows that the adsorption of this gas on the studied adsorbent is complex and involves several types of adsorption sites with different energies and capacities. The Langmuir model with a high coefficient of determination (*R*^2^ ≈ 0.99) describes monolayer adsorption and homogeneous surface to some extent, but the difference in parameters compared to CO_2_ adsorption is due to the molecular characteristics of nitrogen. Dual-site models such as DSL and DS-LF with *R*^2^ close to 1 better reflect the surface heterogeneity and the presence of adsorption sites with different energies. In particular, the *n* parameters in LF model reflect the heterogeneity's intensity and the adsorption energy's distribution. In general, the best description of nitrogen adsorption is related to the two-site and LF models, which will express the complexity of the surface structure and the diverse adsorption mechanisms and are consistent with similar findings in the scientific literature on the importance of surface heterogeneity in the adsorption of nonpolar gases.

The results of fitting the CO_2_ gas adsorption data with different isotherm models (Table 5) indicate the studied adsorbent's complex and multimodal adsorption behavior. The Langmuir model with a high *R*^2^ (more than 0.99) shows that the monolayer adsorption and homogeneous surface are described to an acceptable extent. However, significant fluctuations in the values of the *q* and *k* parameters indicate the heterogeneity of the surface and the diversity of adsorption sites. The DSL model, which has a coefficient of determination close to 1, confirms the existence of two adsorption sites with different capacities and energies. The significant difference between the parameters *q*_1_ and *q*_2_ as well as *k*_1_ and *k*_2_ indicates the different contribution of each site to the adsorption process, especially very large or small values of the equilibrium constants indicating a substantial difference in the adsorption propensity of molecules to each site, which is common in complex and multiphase adsorbents.^57^ The LF model, which is used to describe adsorption on surfaces with heterogeneous energy distribution of adsorption sites, also explains the data well with an *R*^2^ above 0.998; a parameter *n* less than 1 in most cases indicates surface inhomogeneity and non-uniformity of adsorption energy. Finally, the DS-LF model, which combines two adsorption sites with heterogeneous energy distribution, provides the best fit with an *R*^2^ close to 0.999. The values of the parameters *n*_1_ and *n*_2_, which are close to or higher than 1 in some cases, indicate the difference in the intensity of heterogeneity of each site and indicate that the adsorption at each site is differently affected by the surface energies.^58,59^ Therefore, the analysis of the obtained parameters confirms that the gas adsorption on the studied adsorbent does not only involve monolayer adsorption on homogeneous sites, but also there are multiple sites with different energies and significant heterogeneity on the surface, which are best described by the two-site and Freundlich models.

### 3.3. CO_2_/N_2_ selectivity

The preferential adsorption of CO_2_ over N_2_ is considered an essential parameter in evaluating the interaction between gas molecules and nanosorbents. Ideal adsorbed solution theory (IAST) was used to quantify the selectivity between CO_2_ and N_2_. The DSL model was used to fit the isotherm of individual gases. Binary gas selectivity (*i.e.*, CO_2_/N_2_) was calculated using eqn (5):^60^

5 *S* = (*x*_1_/*x*_2_)(*y*_2_/*y*_1_)

**Fig. 12:**
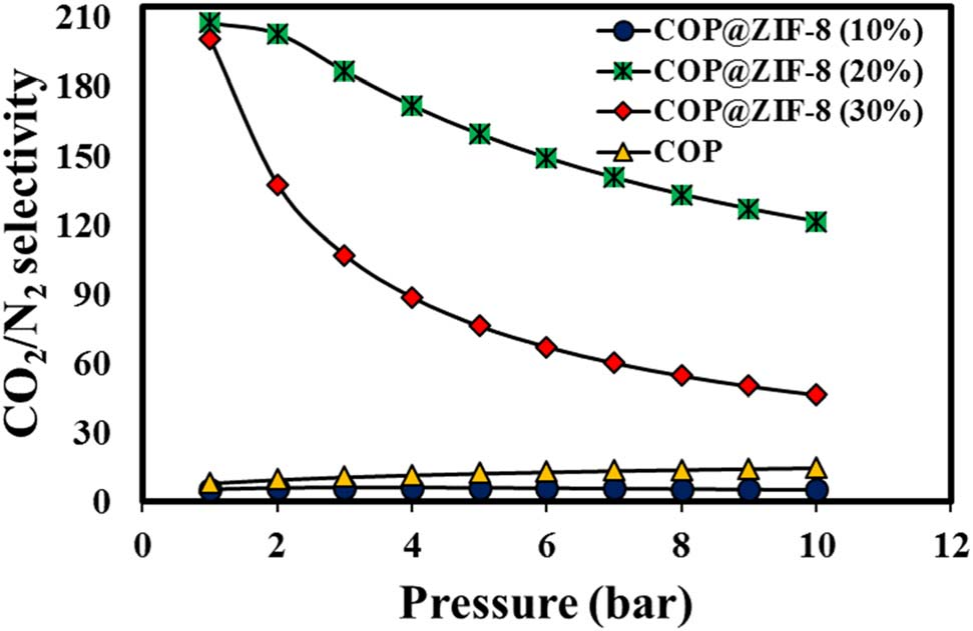
IAST predicted selectivity for CO_2_/N_2_ (15 : 85) of COP@ZIF-8 core–shell.

The parameters *S*, *x*, and *y* represent the selectivity of one gas over another gas, the mole fraction in the solid phase, and the mole fraction in the gas phase, respectively. In general, the selectivity of an adsorbent is determined by the adsorption mechanism employed, whether it is based on chemical or physical adsorption. Chemical interactions between gas molecules and adsorbents are crucial for gas separation when chemical adsorption is the dominant process. In the case of physical adsorption, the physical characteristics of the gas molecule, such as kinetic diameter, polarizability, and dipole moment, should be considered.^61^ The results of CO_2_/N_2_ selectivity (15 : 85) by pure COP nanosorbents, COP@ZIF-8 (10–30%), at 300.15 K temperature are shown in Fig. 12. As the figure shows, the adsorption selectivity of COP@ZIF-8 (20%) and COP@ZIF-8 (30%) nanosorbents is significantly (207.752 and 200.592 in ambient conditions, respectively) improved compared to pure COP (14.824). The presence of amine functional groups in the structure leads to an increase in CO_2_ adsorption capacity and a decrease in N_2_ adsorption capacity. This is due to the more significant interaction between CO_2_ molecules and the amine functions of the adsorbent, which reduces the N_2_ adsorption capacity, which is exclusively controlled by physical adsorption. Another critical factor in this regard is the increase in mesoporosity in the COP@ZIF-8 structure, effectively reducing the physical adsorption of N_2_.

### 3.4. Comparison of the CO_2_/N_2_ selectivity nanosorbents of present study with other sorbents

This study used a very efficient and cost-effective adsorbent with easy and rapid synthesis to achieve CO_2_/N_2_ selectivity. Compared to other adsorbents in the literature, prepared nanosorbents showed a significant amount of adsorption capacity and selectivity (Table 6).

**Table 6:** Comparison of the CO_2_/N_2_ selectivity of prepared nanosorbents compared to other adsorbents in the literature

Sorbent	*T* (K)	*P* (bar)	CO_2_/N_2_ selectivity	Ref.
COP@ZIF-8 (20%)	300.15	1	207.752	This study
COP-1	318	1	25	62
COP-2	318	1	7.9	62
PCN-26	273	1	49	63
CTFs	273	1	31.2	64
Azo-COP-1	263	1	27.4	65
Azo-COP-1	273	1	44.0	65
Azo-COP-1	298	1	30.7	65

## 4. Conclusion

In this research, COP and COP@ZIF-8 (10–30%) core–shell was prepared using the solvothermal method. The synthesized nanosorbents are used for the selective adsorption of CO_2_/N_2_. The results of the COP@ZIF-8 (20%) sample showed the highest gas adsorption capacity (7.271 mmol g^−1^) under a pressure of 10 bar at 300.15 K. The synthesized COP@ZIF-8 core–shell showed excellent surface area (1356.942 m^2^ g^−1^) with a pore volume of 0.734 cm^3^ g^−1^, and mean pore diameter of 2.137 nm. Calculations were done with the IAST method, and it was observed that the COP@ZIF-8 (20%) sample had the highest selectivity rate (207.752 at 300.15 K and 1 bar) among all samples. Therefore, it can be said that COP-based porous nano sorbents are promising adsorbents for the selective adsorption of CO_2_ gas from the CO_2_/N_2_ mixture due to their nitrogen content, high porosity, stability, and high economic efficiency.

## Author contributions

Soheila sharafinia: writing – original draft, writing – review & editing, visualization, project administration, methodology, investigation, funding acquisition, conceptualization, soft-ware. Nedasadat Saadati Ardestani: writing – review & editing. Alimorad Rashidi: software, resources, methodology, project administration. Fereshteh Abbasy: data curation. Pedram Eskandari: investigation, formal analysis.

## Conflicts of interest

The authors declare that they have no known competing financial interests or personal relationships that could have appeared to influence the work reported in this paper.

## Supplementary Material

RA-015-D5RA03873A-s001

## Data Availability

All data generated or analyzed during this study are included in this published article. TGA curves along with plots of CO_2_ and N_2_ adsorption capacities based on various adsorption models. See DOI: https://doi.org/10.1039/d5ra03873a.
