# Supplementary material for: CO2/N2 selectivity with high efficiency using new flexible coordinate organic polymer-based core–shell
Source: RSC Adv. 2025 Aug 19;15(36):29284–99. doi: 10.1039/d5ra03873a (PMC12377163; doi:10.1039/d5ra03873a)
Supplement: RA-015-D5RA03873A-s001 [file RA-015-D5RA03873A-s001.pdf]

Supplementary Information (SI) for RSC Advances.
This journal is © The Royal Society of Chemistry 2025

Supporting Information

CO_2/ N_2 Selectivity with High Efficiency Using New Flexible Coordinate Organic Polymer-based Core-shell

Soheila Sharafinia[1]*, Nedasadat Saadati Ardestani[2], Alimorad Rashidi[2]*, Fereshteh Abbasy[2], Pedram Eskandari[3]

[1]Department of chemistry, Faculty of Science, Shahid Chamran University of Ahvaz, 613578-3151, Ahvaz, Iran

[2]Nanotechnology Research Center, Research Institute of Petroleum Industry (RIPI), 14665-1998, Tehran, Iran

[3]School of Energy Engineering and Sustainable Resources, College of Interdisciplinary Science and Technology, University of Tehran, 1417935840, Tehran, Iran

Corresponding authors:

E-mail addresses: [1]*sharafi.s2014@gmail.com

[2]*rashidiam@ripi.ir

$$n_{ads} = \frac{V_{GC}}{RT}\left(\frac{P_E^g}{Z_E^g} - \frac{P_I^g}{Z_I^g}\right) - \frac{V_{dead}}{RT}\left(\frac{P_E^{He}}{Z_E^{He}} - \frac{P_{1I}^{He}}{Z_I^{He}}\right) \quad \text{S1}$$

Z_E^g = Gas compressibility factor at equilibrium (P_E^g) pressure.

Z_I^g = Gas compressibility factor at initial (P_I^g) pressure.

Z_E^{He} = Helium compressibility factor at equilibrium (P_E^{He}) pressure.

Z_I^{He} = Helium compressibility factor at initial (P_I^{He}) pressure.

V_{dead} = The dead volume of the system.

V_{GC} = The volume of gas cells of the system.

T = The temperature of the adsorption.

R = The ideal gas constant.

Note: the calculations of compressibility factors were done via the Soave-Redlich-Kwong (SRK) equation of the state:

$$Z^3 - Z^2 + \left(\frac{aP - b^2P^2}{R^2T^2} - \frac{bP}{RT}\right)Z = \frac{ab\,P^2}{R^3} \quad \text{S2}$$

$$a = 0.4274\,\frac{R^2T_c^2}{P_c}\left(1 + m\left(1 - \sqrt{T_r}\right)\right)^2 \quad \text{S3}$$

$$b = 0.0866\,\frac{RT_c}{P_c} \quad \text{S4}$$

$$m = 0.48 + 1.574\omega - 0.176\omega^2 \quad \text{S5}$$

ω = The acentric factor.
T_c = Critical temperature.
T_r = Reduced temperature.

1. BET Surface Area Analysis

The BET parameters are obtained by placing the sample at a constant temperature of liquid nitrogen (77 K) and measuring the volume of nitrogen gas adsorbed on the sample surface at different relative pressures. As the pressure is gradually increased, the rate of gas adsorption is recorded, and then, as the pressure is decreased, the gas desorption rate is measured. The data is plotted as an adsorption and desorption isotherm. Then, using the BET equation, which is a linear relationship between the relative pressure P/P0 and the volume of adsorbed gas V, the key parameters of the molecular monolayer volume Vm and the adsorption constant C are extracted. These parameters are obtained from the slope and width from the origin of the BET linear plot. Finally, knowing the molecular volume of the gas, the effective cross-sectional area of an adsorbed molecule (for nitrogen about 0.162 nm^2), and the mass of the sample, the BET-specific surface area is calculated using the formula:

$$S_{BET}=Vm\times N\times a/22400\times m \quad \text{S1}$$

where N is Avogadro's number and m is the mass of the sample. This method allows for the accurate determination of the active surface area of the sample.

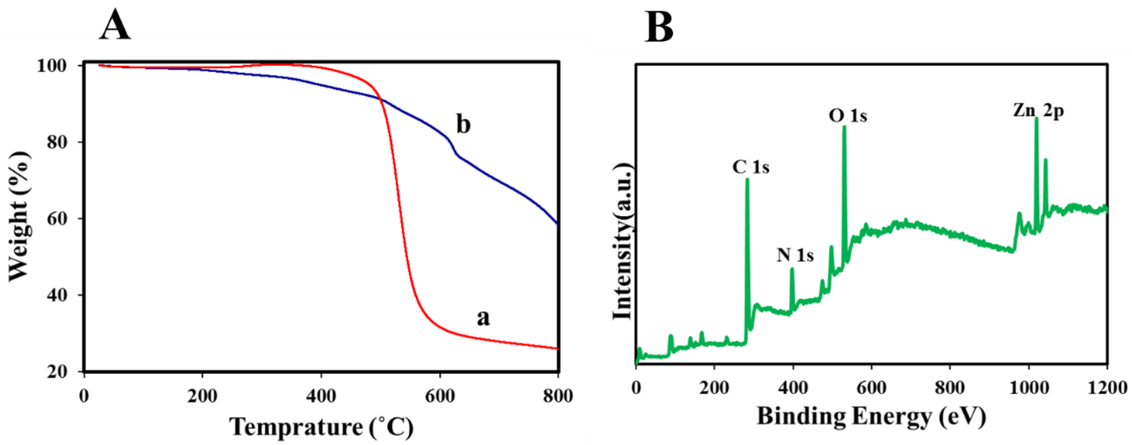

Figure S1. A) TGA curves of a: COP, b: NPC, COP@ZIF-8 core-shell; **B)** XPS full survey spectrum of COP@ZIF-8 core-shell.

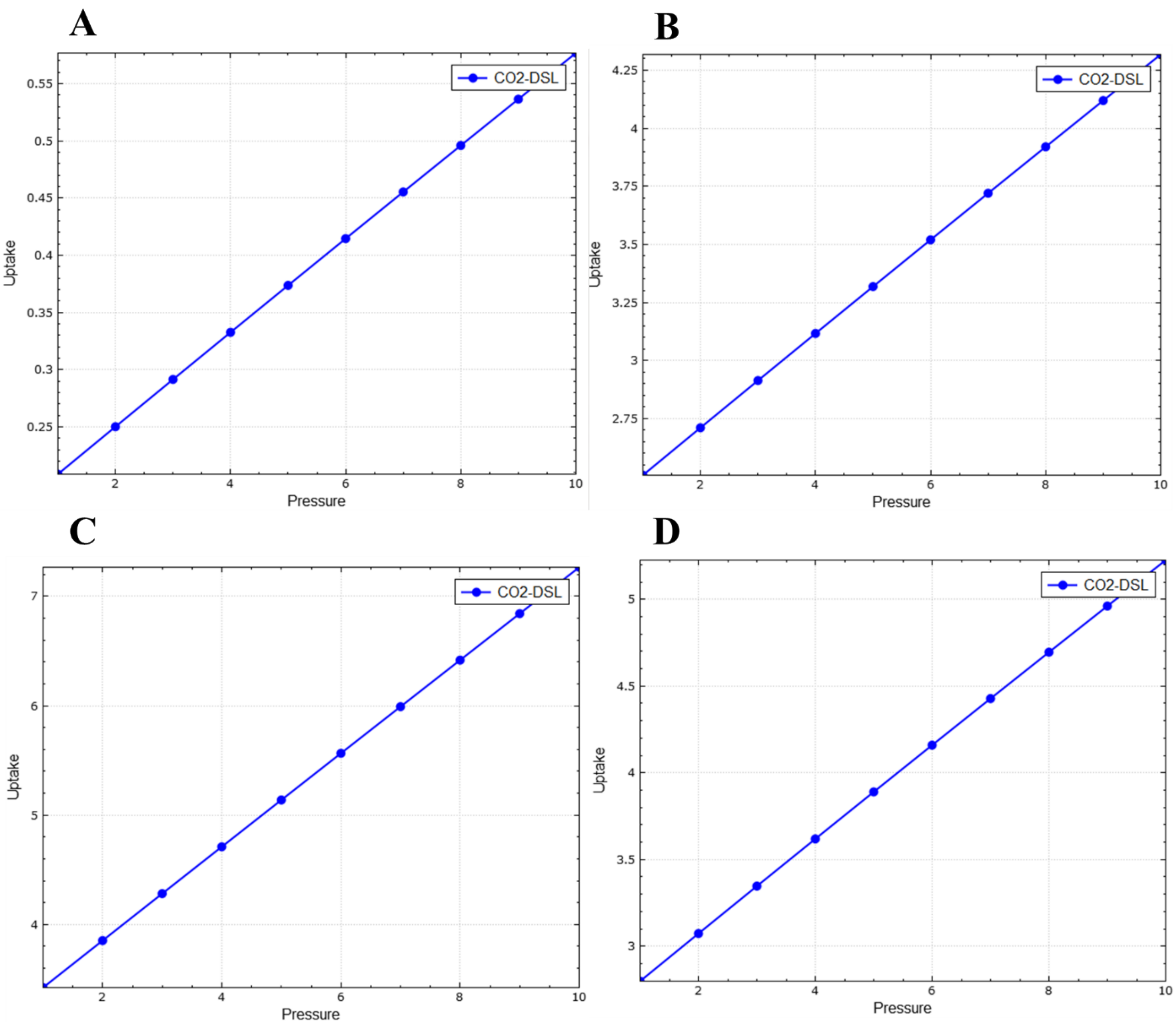

Figure S2. The plots of CO_2 adsorption capacity by DSL model on **A)** COP; **B)** COP@ZIF-8 (10); **B)** COP@ZIF-8 (20); **B)** COP@ZIF-8 (30).

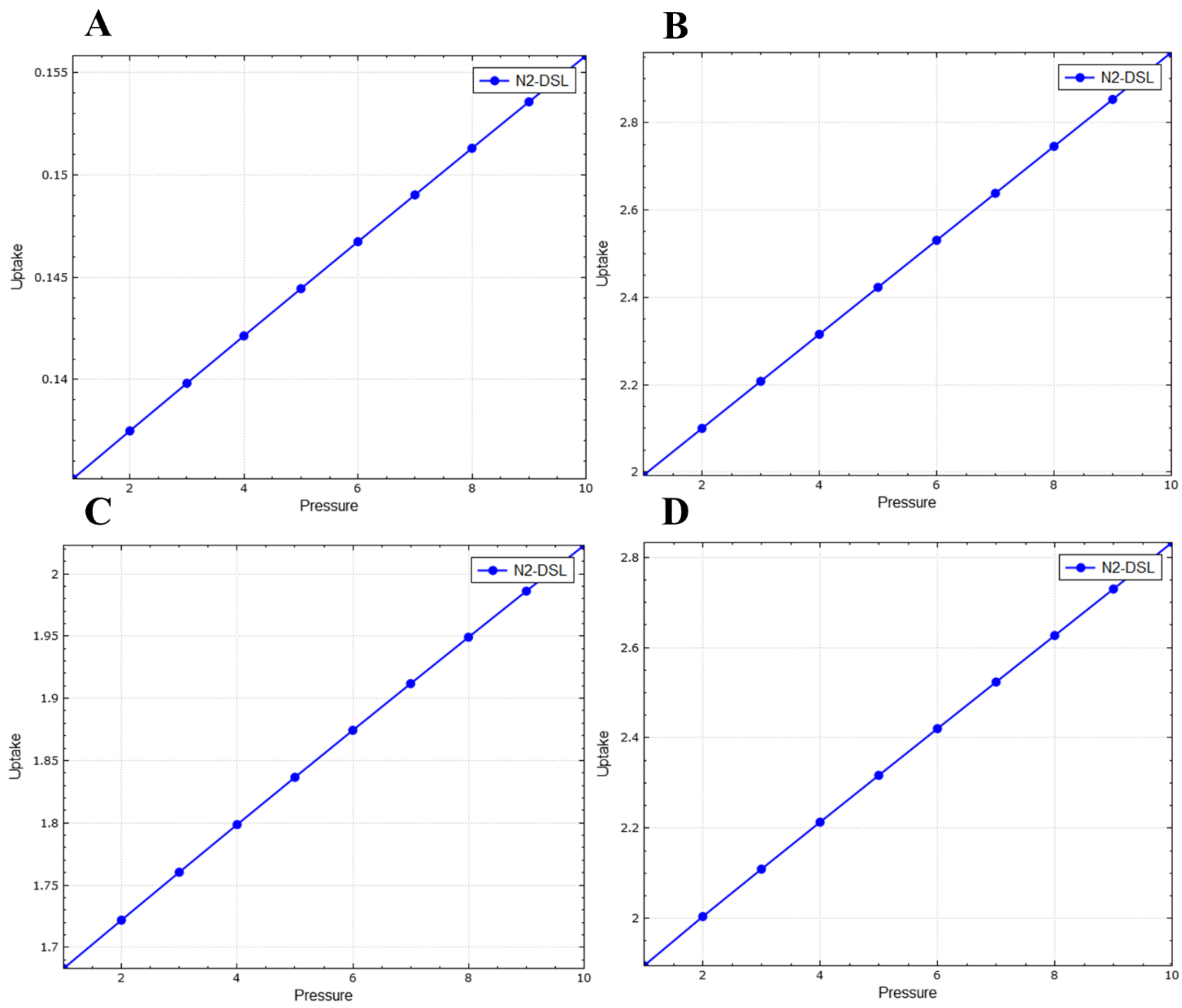

Figure S3. The plots of N_2 adsorption capacity by DSL model on **A)** COP; **B)** COP@ZIF-8 (10); **B)** COP@ZIF-8 (20); **B)** COP@ZIF-8 (30).
